# An ultrathin ionomer interphase for high efficiency lithium anode in carbonate based electrolyte

**DOI:** 10.1038/s41467-019-13783-1

**Published:** 2019-12-20

**Authors:** Yu-Ting Weng, Hao-Wen Liu, Allen Pei, FeiFei Shi, Hansen Wang, Chih-Yuan Lin, Sheng-Siang Huang, Lin-Ya Su, Jyh-Ping Hsu, Chia-Chen Fang, Yi Cui, Nae-Lih Wu

**Affiliations:** 1 Department of Chemical Engineering, National Taiwan University, Taipei, 10617 Taiwan; 2 Advanced Research Center of Green Materials Science and Technology, National Taiwan University, Taipei, 10617 Taiwan; 3 Department of Materials Science and Engineering, Stanford University, Stanford, CA 94305 USA; 4 Department of Chemical Engineering, National Taiwan University of Science and Technology, Taipei, 10617 Taiwan; 5 Industrial Technology Research Institute, Hsin-Chu, 300 Taiwan; 6 Stanford Institute for Materials and Energy Sciences, SLAC National Accelerator Laboratory, 2575 Sand Hill Road, Menlo Park, CA 94025 USA

**Keywords:** Energy storage, Batteries, Materials for energy and catalysis, Nanoscale materials

## Abstract

High coulombic efficiency and dendrite suppression in carbonate electrolytes remain challenges to the development of high-energy lithium ion batteries containing lithium metal anodes. Here we demonstrate an ultrathin (≤100 nm) lithium-ion ionomer membrane consisting of lithium-exchanged sulfonated polyether ether ketone embedded with polyhedral oligosilsesquioxane as a coating layer on copper or lithium for achieving efficient and stable lithium plating-stripping cycles in a carbonate-based electrolyte. Operando analyses and theoretical simulation reveal the remarkable ability of the ionomer coating to enable electric field homogenization over a considerably large lithium-plating surface. The membrane coating, serving as an artificial solid-electrolyte interphase filter in minimizing parasitic reactions at the electrolyte-electrode interface, enables dendrite-free lithium plating on copper with outstanding coulombic efficiencies at room and elevated (50 °C) temperatures. The membrane coated copper demonstrates itself as a promising current collector for manufacturing high-quality pre-plated lithium thin-film anode.

## Abstract (web summary)

The authors here report an ultrathin ionomer membrane as an artificial solid-electrolyte interphase filter that minimizes parasitic reactions and enables stable dendrite-free lithium plating-stripping cycles in a carbonate-based electrolyte. The protected anodes exhibit outstanding coulombic efficiencies at room and elevated (50 °C) temperatures.

## Introduction

Exploring energy storage devices with high energy density has become a crucial topic of interest worldwide. For improving the performance of Li-ion batteries (LIBs), the feasibility of using Li metal as an anode material has been attracting considerable attention owing to its low redox electrochemical potential (−3.040 V vs. a standard hydrogen electrode) and excellent theoretical specific and volumetric capacities of 3860 mAh g^−1^ and 2045 mAh cm^−3^, respectively. These capacities are much higher than those of graphite, a state-of-the-art commercial LIB anode material (370 mAh g^−1^ and 760 mAh cm^−3^, respectively). The use of a Li metal anode enables realization of LIBs with high energy density and less weight. Nevertheless, dendrite formation^1–3^ upon repetitive Li plating–stripping and insufficient coulombic efficiency (CE) are to date the two major problems encountered for realizing Li metal anode technology. Li dendrite could cause short circuiting and eventually lead to fire hazard in practical applications, whereas low CE leads to rapid capacity reduction. To suppress the formation of Li dendrites, numerous studies have focused on using gel polymer and ceramic electrolytes^4–11^. Other studies have focused on designing architectural surface structures for suppressing dendrite on either Li metal anodes or copper (Cu) current collectors^12–15^ with various coating layers of porous nanomaterials, such as carbon nanotubes (CNTs)^16–18^, graphene^19,20^, silica^21,22^. A concept of using functionalized separators to mitigate dendrite formation and enhance cycle stability for Li metal anode has also been proposed^23,24^.

Obtaining nearly 100% CE—the ratio of the Li stripping capacity to the Li plating capacity—is essential for maintaining a long cycle life. Regarding the electrochemical behavior of Li ions, an ideal solid-electrolyte interphase (SEI) layer containing appropriate organic and inorganic products on an electrode surface after the first cycle can effectively prevent further electrolyte degradation and selectively allow only Li ions to pass through during the repeated charging–discharging process. Therefore, the development of a good SEI layer on an electrode surface is critical for cycle stability of LIBs. For an ether-based electrolyte system, an elastic oligomer SEI layer with flexibility can be formed on the Li metal surface through the ring-opening reaction of ether solvents^25^. With adding Li nitride (LiNO_3_) as an additive into the electrolyte, CEs for Li metal plating and stripping cycles can in general be improved to approximately 98%, owing to the formation of a passivating protective layer on the surface of the Li metal anode^26^. However, the most commonly used Li salt, namely, Li bis(trifluoromethanesulfonyl) imide (LiTFSI), in ether-based electrolytes readily decomposes above 3.5 V^27^, thus leading to limited application. By contrast, carbonate-based electrolytes have been widely applied in commercial LIBs because of their compatibility with electrode materials and satisfactory stability over a substantially higher electrochemical range (>4 V). However, the CE of the Li metal anode in typical carbonate-based electrolytes barely reaches 96%, and thus some studies have explored new types of additives^28–35^ and different electrolyte compositions^36–38^, aiming to improve CE. In particular, addition of fluoroethylene carbonate (FEC) has been reported to be beneficial for improving the cycle stability and CE for Li metal anode in carbonate-based electrolytes^32,33^.

The formation of an artificial SEI (ASEI) through a surface coating of polymer, such as poly(vinylidene difluoride)^39^, silly putty^40^, poly((N-2,2-dimethyl-1,3-dioxolane-4-methyl)-5-norbornene-exo-2,3-dicarboximide)^41^, poly(dimethylsiloxane)^42^, and styrene butadiene rubber^43^, on the Cu foil surface has been suggested for suppressing Li dendrite formation. Particularly, cation-selective ionomer membranes, which contain negatively charged groups, such as –SO_3_^−^, –COO^−^, and –PO_3_^2−^, that are fixed to the polymer backbone, are promising ASEI candidates because they have the potential of simultaneously facilitating the passage of Li ions^44,45^ and enabling the high CEs by reducing direct contact between a solvent and the anionic ions of a salt with Li metal anode, thereby reducing solvent or anion decomposition. Li dendrite suppressing enabled by cation ionomer membranes in the literature has so far been attributed mainly to the high transference number, t_+_, of the ionomer, which prolonged the so-called Sandy’s time for the occurrence of dendritic plating^45,46^. However, the theory was built upon metal plating in a homogeneous electrolyte without taking into account of the electrolyte–ionomer membrane interface. In fact, several non-ionomer membranes have also been reported to mitigate Li dendrite formation^39–42^.

For the development of Li metal anode, surface modification layers with thicknesses ranging from hundreds to a few microns^39–43^ have been reported in the literature. Clearly, to truly capitalize on the high energy density obtained using Li metal anode, the thickness of any electrode surface modification layer on either Li or Cu should be kept to its minimum value. Tu et al.^45^ once demonstrated an ASEI fabricated using a 200-nm-thick fluorine-based cation ionomer membrane that was effective in suppressing Li dendrite formation in a carbonate-based electrolyte. However, the CE was 80%, and it was improved to 92% only by using an additional thick Al_2_O_3_ filter membrane (Whatman). Other than enabling high CEs, a thin ASEI layer should require sufficient mechanical and thermal robustness that is essential for sustaining prolonged cycling.

**Fig. 1:**
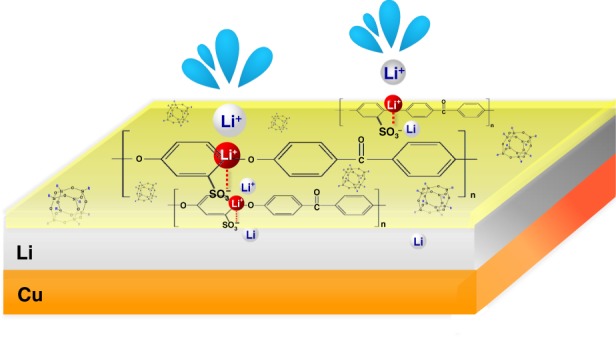
**Ionomer interphase for effective Li metal anodes.** Schematic showing Li plating and stripping on the SPEEK-Li/POSS-coated Cu: solvated Li ions undergo desolvation and diffusing across the cation-selective membrane to form a uniform and dense Li metal layer, which is subsequently removed, in between the membrane and Cu surface with a high CE and excellent long-term cycling stability.

In this study, we successfully fabricate an ultrathin (≤100 nm) ASEI layer for Li and Li-free anodes that are effective in suppressing Li dendrite formation while effectively improving CEs in the carbonate-based electrolyte for extended cycling at both room and elevated (50 °C) temperatures. The ASEI, referred to as SPEEK-Li/POSS, is an ionomer membrane based on sulfonated polyether ether ketone (SPEEK) exchanged with Li ions (Li-SPEEK; Supplementary Fig. 1a) and embedded with a polyhedral oligosilsesquioxane (POSS; Supplementary Fig. 1b). Compare to the commercial fluorine-based (CF-) cation ionomer membrane (such as Nafion®), SPEEK possessed relatively higher mechanical strength and dimensional rigidity due to its aromatic components. In addition, the Li-ion conductivity of SPEEK is easily increased through sulfonation followed by Li-ion exchange using titration. Cage-like POSS compounds have been used as versatile inorganic fillers for modifying various properties of the polymer hosts due to their unique hollow rigid structures and inorganic cube-octameric siloxane skeletons. In contrast to conventional ceramic oxide nanoparticles, which often suffer from dispersion problem for preparing composite materials, POSS can readily and homogenously blend with SPEEK. As schematically represented in Fig. 1, for Li plating and stripping on the SPEEK-Li/POSS-coated Cu, Li ions diffuse across this cation-selective membrane to form a uniform and dense Li metal layer, which is subsequently removed, in between the membrane and Cu surface with a high CE and excellent long-term cycling stability even for high Li plating capacity (e.g., 3 mAh cm^−2^) in both carbonate- and ether-based electrolytes. Furthermore, the full-cell configuration of Cu|Li|SPEEK-Li/POSS||LiFePO_4_ (LFP) presentes considerably lower polarization than the cell without the coating and demonstrates remarkable rate and cycle performance.

## Results

### Characterizations of ionomer membrane

**Fig. 2:**
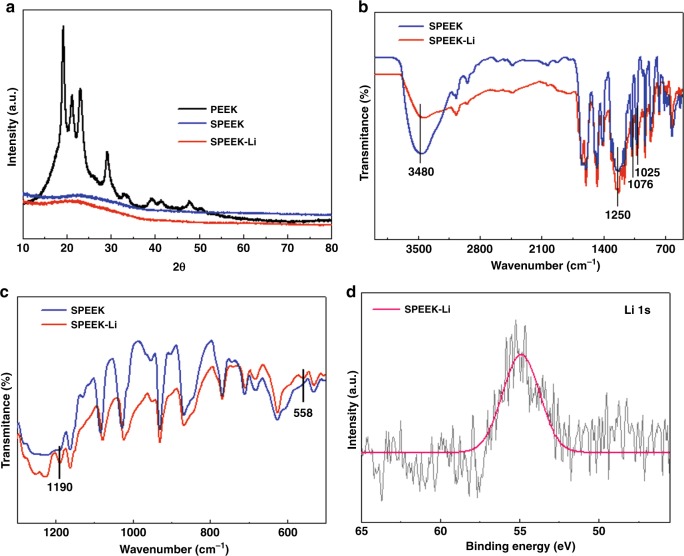
Spectroscopic characterizations of SPEEK-Li. **a** XRD pattern; FTIR spectra of SPEEK and SPEEK-Li within different wavenumber ranges, **b** 4000–500 cm^−1^ and **c** 1300–500 cm^−1^; **d** XPS spectrum of Li in SPEEK-Li.

The synthesis of SPEEK-Li ionomer was subjected to the characterizations of X-ray diffraction (XRD), X-ray photoelectron spectroscopy (XPS), and Fourier transform infrared (FTIR) spectroscopy. Generally, the amorphous polymer is more favorable to ionic conduction than crystalline structure^47^. XRD analysis revealed that the highly crystalline PEEK turned amorphous after sulfonation (SPEEK) and Li-ion exchange (SPEEK-Li) (Fig. 2a). The sulfonate group of SPEEK was confirmed by FTIR (Fig. 2b). The FTIR results presented absorption bands at 1250 and 1076 cm^−1^ that were attributed to asymmetric and symmetric sulfur–oxygen stretching vibrations (O=S=O), respectively. Moreover, the absorption band at 1025 cm^−1^ was attributed to S‒O stretching. The elemental analysis provided a sulfur-to-carbon weight ratio corresponding to nearly 85% sulfonation. Compared with the SPEEK spectrum, the intensity of the absorption band at 3480 cm^−1^ of SPEEK-Li considerably decreased because of the reduced O‒H, and the new bands observed at 558 and 1190 cm^−1^ were assigned to O‒Li‒O and antisymmetric Li‒O‒Li stretching, respectively, (attributable to the clustering of polar ionic groups; Fig. 2c)^48^. Furthermore, XPS analysis revealed a characteristic binding energy of 55 eV for Li^+^ (Fig. 2d). The FTIR and XPS results confirmed successful exchange of protons for Li ions at the sulfonate groups.

**Fig. 3:**
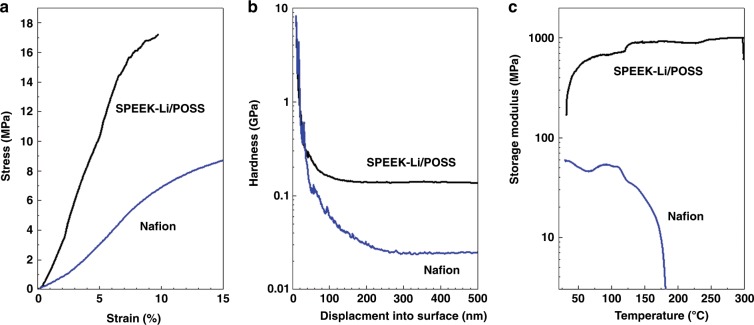
Characterizations of mechanical properties. **a** Tensile stretch test; **b** nanoindentation test; **c** DMA test on free-standing SPEEK-Li/POSS and Nafion® membranes.

SPEEK-Li/POSS was prepared by dissolving SPEEK-Li and POSS in a solvent (see “Methods”) followed either by solution casting for free-standing membranes or by tape casting for coatings. Scanning electron microscopy (SEM) analysis indicated uniformly dispersed POSS submicron clusters embedded within the polymer matrix (Supplementary Fig. 2). Mechanical tests were conducted using nanoindentation and dynamic mechanical analyses (DMA) on the free-standing membranes. A Nafion® membrane, a commonly known cation ionomer, was also fabricated by the same method for comparison. The SPEEK-Li/POSS membrane showed a tensile strength of 17 MPa and exhibited a storage modulus an order of magnitude higher and a hardness value more than five times greater than those of the Nafion® membrane (Fig. 3a, b). It was also found (Supplementary Fig. 3) that, compared with SPEEK-Li, SPEEK-Li@POSS had a higher tensile strength by nearly 30% and exhibited substantial improvement in flexibility and stretchability with increasing fracture strain by more than fivefolds (from 1.7 to 9.6%). The SPEEK-Li/POSS membranes possessed considerably superior mechanical strength and dimensional rigidity than the fluorine-based membrane over a wide temperature range (Fig. 3c). Furthermore, cross-section SEM analysis indicated the thicknesses of the membrane coatings on Cu were in the range of 95–100 nm (Supplementary Fig. 4). Electrochemical measurements on the free-standing SPEEK-Li/POSS membranes showed an ionic conductivity of 1.6 × 10^−4^ S cm^−1^ (Supplementary Fig. 5) and a transference number of 0.73 (Supplementary Fig. 6).

### Operando and postmortem analyses on Li plating

**Fig. 4:**
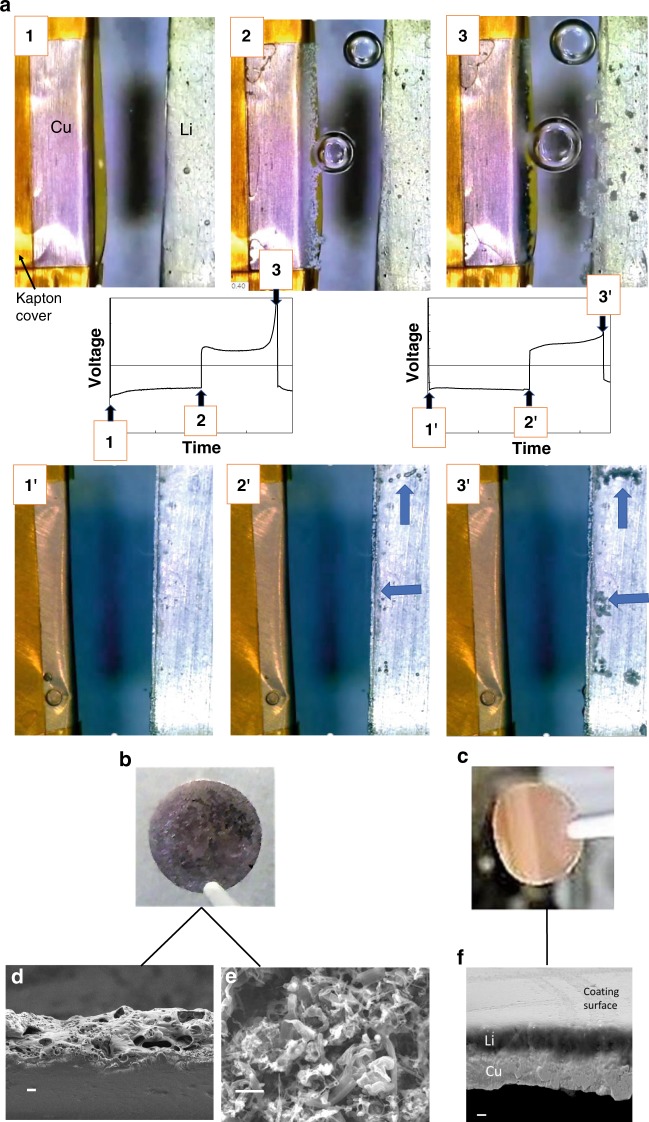
Operando and postmortem analyses on Li plating. **a** Operando observation of Li-plating and stripping in a glass cell using (frames 1–3) a pristine Cu electrode and (frames 1′–3′) a SPEEK-Li/POSS-coated Cu electrode as anodes and the Li metal foil as a cathode with a carbonate-based electrolyte; the voltage-vs.-time plots indicate the moments when the snapshots are taken; optical images showing the Li-plating morphology for **b** a pristine Cu electrode and **c** a SPEEK-Li/POSS-coated Cu electrode which have been subjected to two and half galvanostatic Li plating–stripping cycles in coin cells (current density = 1 mA cm^−2^ and Li plating capacity = 2 mAh cm^−2^); **d** SEM micrograph showing the cross-section of (**b**), scale bar: 10 μm; **e** SEM micrograph showing the surface morphology of (**b**) after being washed with organic solvent, scale bar: 5 μm; **f** SEM micrograph showing the cross-section and surface morphology of (**c**), scale bar: 5 μm.

To monitor the effect of SPEEK-Li/POSS membrane coating on the Li plating–stripping behaviors in real time, glass electrochemical cells (schematics shown in Supplementary Fig. 7) comprising a Li foil electrode and a folded Cu foil electrode were employed. Figure 4a displays the snapshots acquired using optical microscope (OM) during the first Li plating–stripping cycle (1 mAh cm^−2^). Li plating on the bare Cu electrode caused the formation of a gray deposition layer at the front edge of the folded Cu electrode (frame 2, Fig. 4a). The growing surface of the deposited layer was full of dendritic protrusion. Meanwhile, pits formed on the Li surface due to Li loss. Upon Li stripping from the Cu electrode (frame 3, Fig. 4a), the deposit on the front edge of the Cu electrode partially retracted and turned black, whereas mossy Li formed on the edges of the Li electrode. The black leftover on the Cu electrode is believed to be composed of mainly the carbonaceous SEI materials resulting from the decomposition of electrolyte. By great contrast, for the SPEEK-Li/POSS-coated Cu electrode (frames 1′–3′), no obvious change took place on the Cu electrode; a uniform Li layer that appeared transparent under optical microscopy was deposited in between the membrane and Cu surface and was subsequently stripped. No Li dendritic layer was observed. The occurrence of the Li stripping and plating processes were indicated by the applied constant current and reflected by the pit formation and mossy deposits on the Li surface (for example, arrows in frames 2′ and 3′).

In addition to the operando OM analysis, a parallel postmortem analysis was conducted on the Cu electrodes that had been subjected to galvanostatic Li plating–stripping against a Li metal electrode in coin cells. At the end of the Li-plating stage of the third cycle (2 mAh cm^−2^ at a current density of 1 mA cm^−2^), the electrodes were removed from the cells for analysis. The pristine Cu electrode was found to be black (Fig. 4b), whereas the SPEEK-Li/POSS-coated electrode (containing deposited Li metal) showed a clear Cu luster (Fig. 4c), which indicated substantial reduction in SEI formation and considerable transparency of the deposited Li layer. SEM analysis on the cross-section of the cycled pristine Cu electrode showed the coverage by a thick and ill-defined SEI layer (Fig. 4d). Washing the electrode with organic solvent revealed dendritic Li deposit (Fig. 4e), By great contrast, the cycled SPEEK-Li/POSS-coated Cu electrode contained a uniform and dense Li metal layer sandwiched between the membrane and the Cu foil (Fig. 4f). These results accorded with those of the operando OM analysis.

**Fig. 5:**
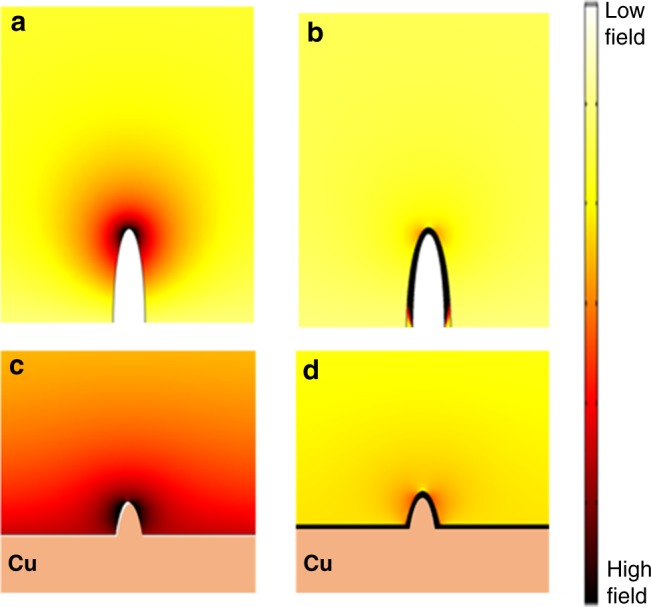
Theoretical simulation on electric-field distribution for electrochemical Li deposition on protuberance-type electrodes. **a** Color-coded electric-field distribution near a bare half-ellipsoidal electrode that mimics the folded electrode in operando optical microscopy analysis, showing concentrated field near the tip of the electrode; **b** the same type of electrode as (**a**) except for having a cation-selective membrane coating, showing strong electric field within the membrane uniformly distributed along the electrode surface; **c** electric-field distribution on a planar electrode with a surface protuberance, showing considerably stronger field near the protuberance tip than on the flat surface; **d** the same type of electrode surface as (**c**) except for having a cation-selective membrane surface coating, showing enhanced field uniformity over the entire electrode surface.

The operando OM analysis shown in Fig. 4a revealed the remarkable ability of a nanometer-thick ionomer membrane coating in changing the Li plating behavior over an area with dimensions orders of magnitude larger (hundreds of micrometers). To our best knowledge, this is by far the first observation reported for such a phenomenon. Theoretical simulation was undertaken to understanding the mechanism for the membrane-homogenized Li plating process. As shown in Fig. 5, a case of Li deposition on a cone-shaped substrate that mimicked the folded Cu substrate in the operando OM analysis was considered (Supplementary Methods: Simulation). The effect of a cation-selective membrane coating on the electric field was examined. In the case of no coating, the electric field, which depicted the trajectories of moving cations, was found to concentrate at the cone tip (Fig. 5a). This is consistent with the OM observation that showed focused Li deposition at the folding edge of the Cu electrode (frame 2, Fig. 4a). In contrast, in the case of the cone surface coated with a cation-selected membrane, because the diffusivity of Li ion within the polymer membrane is substantially lower than that in the bulk liquid electrolyte, diffusion of Li ion across the membrane suffers from a fast potential drop, due to resistance, over a short distance. As electric-field intensity is proportion to the potential gradient, the field is much stronger within the membrane than in the bulk electrolyte. The electric field exhibited a considerably uniform distribution along the entire cone surface (Fig. 5b). This explained the uniform Li deposition observed on the membrane-coated electrode (frame 2′, Fig. 4a).

The implication of the field-homogenization effect on the operation of a planar Cu or Li metal anode is that it may homogenize the electric-field distribution around protuberances on an uneven anode surface to mitigate Li dendrite formation. To illustrate such an effect, the same electric-field calculation was carried out on a flat electrode containing a cone-shape protuberance. As shown in Fig. 5c, the bare electrode shows considerably stronger field near the protuberance tip than on the flat surface. This may lead to preferential Li deposition at the protuberance tip, hence facilitating dendrite formation. By contrast, the presence of the cation-selective membrane coating effectively reduces the field intensity at the tip (Fig. 5d) and enhances field uniformity over the entire electrode surface so that Li may be uniformly deposited without dendrite formation. This is in agreement with the postmortem analysis results (Fig. 4e, f).

The operando OM analysis shown in Fig. 4a also indicated remarkable difference in gas (bubble) formation in these cells. The gaseous species were generated as a result of electrolyte decomposition reactions occurring at the electrode–electrolyte interface. As shown, the gas (bubble) formation was considerably more extensive for the bare Cu electrode (frames 1–3) than for the SPEEK-Li/POSS-coated Cu electrode (frames 1′–3′). This observation is consistent with the difference in the amount of SEI residue observed on the cycle Cu electrodes (Fig. 4b, c). For the bare Cu electrode, the deposited Li is in the dendritic form (Fig. 4e). Dendritic Li possesses a considerably high surface area, which leads to extensive electrolyte decomposition, i.e., gas formation, at its surface. In contrast, in the case of SPEEK-Li/POSS-coated Cu electrode, the extent of the decomposition reaction is reduced on two accounts: first, the coating reduced the amount of solvent molecules in directly contact with the deposited Li; secondly, the deposited Li on the coated Cu electrode has a planar and compact morphology (Fig. 4f), which has a much lower surface area than the dendritic Li in contact with the electrolyte.

**Fig. 6:**
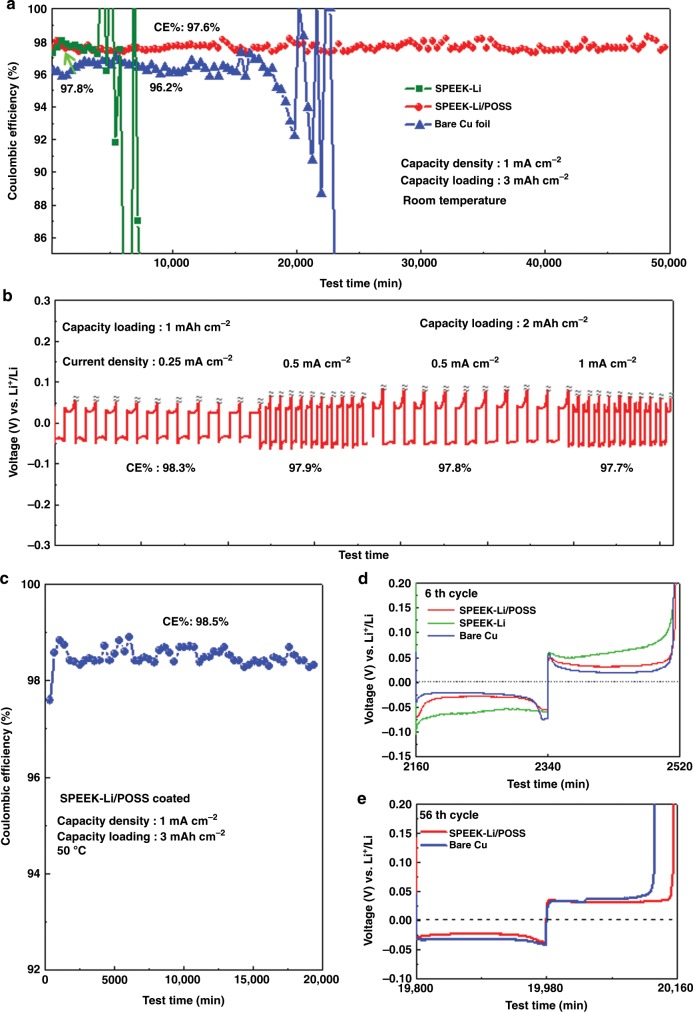
Electrochemical characterizations of Li–Cu cells with the carbonate-based electrolyte. **a** Cycling performance of a bare Cu, and SPEEK-Li and SPEEK-Li/POSS-coated Cu electrodes at 1 mA cm^−2^ with a Li plating loading of 3 mAh cm^−2^ (coulombic efficiency, CE, is defined as the ratio of the Li stripping capacity to the Li plating capacity); **b** voltage profile of SPEEK-Li/POSS-coated Cu cycled at different current densities and Li plating loadings; **c** cycling stability of SPEEK-Li/POSS-coated Cu at 1 with 3 mAh cm^−2^ at 50 °C; **d** voltage profiles for the selected Cu electrodes at the sixth cycle for the test shown in (**a**); **e** voltage profiles for the selected Cu electrodes at the 56th cycle. All CEs indicated are average values over the cycles having the same conditions.

Electrochemical characterizations of the Li plating–stripping behaviors on Cu electrodes were carried out using a Li–Cu configuration having a Li foil as the counter electrode in two electrolyte systems—an ether-based electrolyte (1 M LiTFSI in 1:1 DOL:DME with 3 wt% LiNO_3_) and a carbonate-based electrolyte (1 M LiPF6 in 3:2 EC:DMC with 10 vol% FEC). For the ether-based electrolyte, both bare and the SPEEK-Li/POSS-coated Cu cells exhibited CEs in the range of 98.2–99.2% for wide ranges of current density (0.25–1 mA cm^−2^) and Li-plating capacity loading (1–4 mAh cm^−2^) (Supplementary Fig. 8), similar to those reported in the literature. In contrast, considerable differences were found for the two electrodes in the carbonate-based electrolyte; the bare Cu cell performed significantly worse than the membrane-coated electrodes (Fig. 6a). For example, the bare Cu cell exhibited a CE of only 96.2% at 1 mA cm^−2^ and abruptly failed due to accelerated CE deterioration. The application of the SPEEK-Li/POSS ionomer membrane coating substantially improved the CE to 97.7% and enhanced the cycle stability (Fig. 6a). Overall, the SPEEK-Li/POSS-coated Cu cell exhibited CEs within the range of 97.7–98.3% over wide ranges of current density (0.25–1 mA cm^−2^) and capacity loading (1–3 mAh cm^−2^) (Fig. 6b) in the carbonate-based electrolyte. The remarkable difference in cycle performance between these two electrodes in the carbonate-based electrolyte may be understood based on the analyses shown in Fig. 4. The bare Cu electrode suffers from extensive Li dendrite formation and fast SEI accumulation. By contrast, the Li deposition on coated electrode is in planar form with substantially reduced SEI.

The CE and stable cycling time for the SPEEK-Li/POSS-coated cells shown here are among the highest levels reported for Li plating–stripping on Li-free anodes in carbonate-based electrolytes (Supplementary Table 1). Moreover, the thermally robust nature of the SPEEK-Li/POSS membrane (Fig. 3c) enabled the coated electrode to exhibit outstanding cycle stability for several hundred hours even at 50 °C with high CEs near 98.5% (Fig. 6c). This is the first time a polymeric ASEI has been shown to possess sustainable cycling capability for Li plating–stripping at such a high temperature.

Also worth mentioning is that the SPEEK membrane without POSS improved the CE but was unable to provide long-term cycle stability; the SPEEK cell exhibited an initial CE of 97.8% but failed abruptly after several cycles (Fig. 6a). The failure may be attributed to the occurrence of pinholes or cracks within the membranes, which have been observed in the operando glass-cell experiments. The addition of POSS was therefore crucial for enhancing the mechanical stability of the SPEEK membrane under the electrochemical process in electrolyte. Close examination of the voltage profiles (Fig. 6d, e) revealed that the SPEEK-Li cell suffered from higher polarization, thus suggesting a higher resistance than that in the SPEEK-Li/POSS cell. The open structure of the POSS filler may have facilitated the diffusion of Li ions within the SPEEK-Li matrix. Furthermore, the polarization of the SPEEK-Li/POSS cell remained essentially unchanged after prolonged cycling, whereas the polarization of the pristine Cu cell increased rapidly with cycling.

## Discussion

**Fig. 7:**
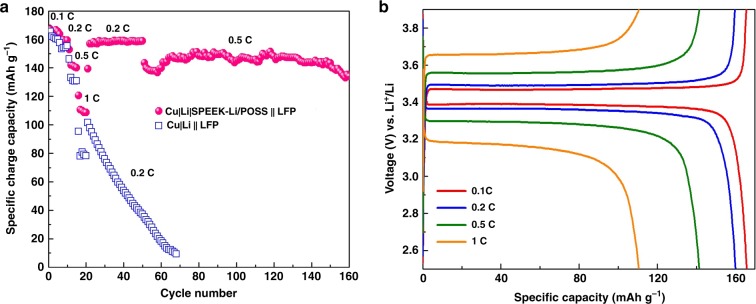
Full-cell electrochemical characterizations with the carbonate-based electrolyte. **a** Comparison in rate and cycle performance between full cells with (Cu|Li|SPEEK-Li/POSS||LFP; red) and without (Cu|Li||LFP; blue) a SPEEK-Li/POSS coating on their Li thin-film anodes. The cathode is LiFePO_4_ (1.85 mAh cm^−2^) and the Li thin-film anode is pre-coated on Cu (loading = 3 mAh cm^−2^). 1 C corresponds to 170 mAh g^−1^ based on LFP in cathode. **b** Corresponding voltage profiles of the Cu|Li|SPEEK-Li/POSS||LFP cell at 0.1, 0.2, 0.5, and 1 C rates.

Thin Li anodes are indispensable for achieving LIBs of high energy density. While mechanical processes, such as calendaring and extrusion, for making thin Li foil are of high cost, electrochemical plating of Li onto suitable current collector, such as Cu, can be a cheaper alternative. The present SPEEK-Li/POSS-coated Cu current collector offers the opportunity for making dendrite-free high-quality pre-lithiated thin-film anodes with minimum SEI contamination. To demonstrate such a potential application, Li thin-film anodes were prepared in coin-cells where Cu current collectors, either with or without the SPEEK-Li/POSS coating, were electrochemically plated against Li foils in the carbonate electrolyte with Li plating loading of 3 mAh cm^−2^, corresponding to approximately 15 μm in thickness. The ionomer coating can have direct positive impact on the quality of the Li thin-film anode. The pre-lithiated anodes were matched against commercial high-loading LiFePO_4_ (LFP; 10.9 mg cm^−2^ or 1.85 mAh cm^−2^) cathodes in the full-cell configuration. As shown in Fig. 7, the cell with the ionomer coating, denoted as Cu|Li|SPEEK-Li/POSS||LFP, demonstrated far superior rate capability and cycle stability than that without the coating (Cu|Li||LFP). The former exhibited a specific capacity of 112 mAh g^−1^ (or 1.2 mAh cm^−2^) at 1 C-rate (1 C = 170 mA g^−1^), as opposed to 80 mAh g^−1^ for the latter. The Cu|Li|SPEEK-Li/POSS||LFP cell exhibited low polarization over a wide range of current density from a 0.1 to 1 C rate (Fig. 7b) and demonstrated long-term cycling stability at a high current density of 0.5 C rate (Fig. 7a).

In summary, a nanometer-thick (≤100 nm) Li-ion ionomer membrane, SPEEK-Li/POSS, containing Li-exchanged SPEEK as the main chain and POSS as the filler was demonstrated to be an efficient ASEI in enhancing the electrochemical performance of Li metal anode. Experimental results revealed its mechanical and thermal robustness and remarkably capabilities in enabling homogenous Li plating and minimizing parasitic reactions at the electrolyte–electrode interface. The Cu|SPEEK-Li/POSS ||Li cells exhibited efficient and stable Li plating/stripping cycles without dendrite formation in both ether- and particularly carbonate-based electrolytes. Specifically, the ionomer membrane coating enabled remarkable enhancement in CE up to 98.5% under high current density and Li plating loadings (1 mA cm^−2^ for 3 mAh cm^−2^) while cycling for hundreds of hours at both room and elevated (50 °C) temperatures in a carbonate-based electrolyte. When SPEEK-Li/POSS-coated Cu was applied in a full-cell, Cu|Li|SPEEK-Li/POSS||LiFePO_4_, consisting of a Li thin-film anode against a commercial high-loading LiFePO_4_, the electrochemical performance of cell demonstrated substantially enhanced rate capability and cycle stability.

## Methods

### Preparation of SPEEK-Li

Poly (ether ether ketone) (PEEK) pellets were purchased from Aldrich and all chemical reagents used in this study were of an analytical grade. For the sulfonation of PEEK (SPEEK), 1 g of PEEK was dissolved in 19 ml of concentrated (98%) sulfuric acid (H_2_SO_4_) and was vigorously stirred at 40 °C until complete dissolution. After 4 h, the solution was poured into a large excess of ice water to precipitate the polymer. And then, the pre-lithiation of SPEEK (SPEEK-Li) could be obtained by dropping 1 M lithium hydroxide (LiOH) into the polymer precipitation until the pH value became neutral. After filtration, the polymer was finally dried in an oven under 90 °C for 24 h.

### Preparation of SPEEK-Li@POSS coating electrode

To prepare the polymer coating working electrode, SPEEK-Li and polyhedral oligosilsesquioxane (POSS, Sigma-Aldrich) with weight ratios of 80/20 in dry basis were mixed with N,N-dimethylacetamide (DMAc, 99.5%, Sigma-Aldrich) to produce the polymer solution, which was then casted into a thin-film on a Cu foil and dried at 60 °C for 20 h in a vacuum oven.

### Material characterizations

Powder XRD (Ultima IV, Rigaku) was used to examine the crystallinity of precursor PEEK after sulfonation and pre-lithiation. The functional groups were confirmed by FTIR spectroscopy (Perkin Elmer Spectrum 100). The FTIR spectra for SPEEK-Li after cycling were measured using a Nicolet iS50 FT/IR Spectrometer (Thermo Fisher) with a diamond attenuated total reflectance attachment in an Ar-filled glovebox. Li content in SPEEK-Li was verified by XPS (Thermo Scientific, Theta Probe). The morphologies of Li deposition on the Cu foil surface with/without SPEEK-Li@POSS coating were examined via SEM (JEOL JSM-7600F). And the SEM samples were prepared in an Ar-filled glovebox and transferred into the SEM chamber using an argon-filled desiccator.

### Electrochemical characterizations

To test electrochemical properties, CR2032-type coin cells were assembled in an argon-filled glove box with lithium foil as counter and references electrodes. The pristine and modified Cu foils were punched into 12-mm-diameter disks as the working electrodes. A commercial polypropylene separator (Celgard 2400) was placed between the two electrodes. A separator ring with 12-mm inner diameter was placed around the working electrode to reduce unfavorable Li deposition outside of the working electrode surface. The ether-based electrolyte was composed of 1 M lithium bis(trifluoromethanesulfonyl)imide (LiTFSI, 99.95%, Sigma-Aldrich) in a 1:1 (v/v) mixture of 1,3-dioxolane (DOL, 99.8%, Sigma-Aldrich) and 1,2-dimethoxyethane (DME, 99.5%, Sigma-Aldrich) with 3 wt% lithium nitrate (LiNO_3_) as an additive. And carbonate based electrolyte was 1 M lithium hexafluorophosphate (LiPF_6_, 99.99%, Sigma-Aldrich) in a 3:2 (v/v) mixture of ethylene carbonate (EC, 99%, Sigma-Aldrich) and dimethyl carbonate (DMC, ≥99%, Sigma-Aldrich) with 10 vol% FEC (98%, Alfa Aesar) as an additive. Li plating/stripping performance tests were carried out with various current densities of 0.25, 0.5, 1, and 2 mA cm^−2^ with a fixed Li capacity loading of 1 mAh cm^−2^ onto the working electrode, and the galvanostatic charge–discharge tests were performed with a cut-off voltage range from 0 to 1.0 V (vs. Li/Li^+^) by using the Maccor/Series 4000 battery testing system. Current was applied in a galvanostatic mode with a current density of 1, 2, and 5 mA cm^−2^, respectively. For the application of full-cell, a LiFePO_4_ electrode (Advanced Lithium Electrochemistry Co., Ltd.; Aleees, the mass loading of LFP was 10.9 mg cm^−2^) was used as cathode and the electrolyte was 1 M LiPF_6_ in EC/DMC (3/2 in vol%) with 10 vol% FEC. For comparing, Li foil and Li coated with SPEEK-Li@POSS were used as anode, respectively. Galvanostatic charge–discharge tests were performed with a cut-off voltage range of 2.5–3.9 V (vs. Li^+^/Li) by using the Maccor/Series 4000 battery testing system. The specific capacity was expressed based on the weight loading of LiFePO_4_.

## Supplementary information

Supplementary Information

## Data Availability

The authors declare that the data supporting the findings of this study are available within the paper and its Supplementary information file.
