# Supplementary material for: An ultrathin ionomer interphase for high efficiency lithium anode in carbonate based electrolyte
Source: Nat Commun. 2019 Dec 20;10:5824. doi: 10.1038/s41467-019-13783-1 (PMC6925282; doi:10.1038/s41467-019-13783-1)
Supplement: Supplementary file 1 — Supplementary Information [file 41467_2019_13783_MOESM1_ESM.pdf]

SUPPLEMENTARY INFORMATION

An Ultrathin Ionomer Interphase for High Efficiency Lithium Anode in Carbonate Based Electrolyte

Weng *et al.*

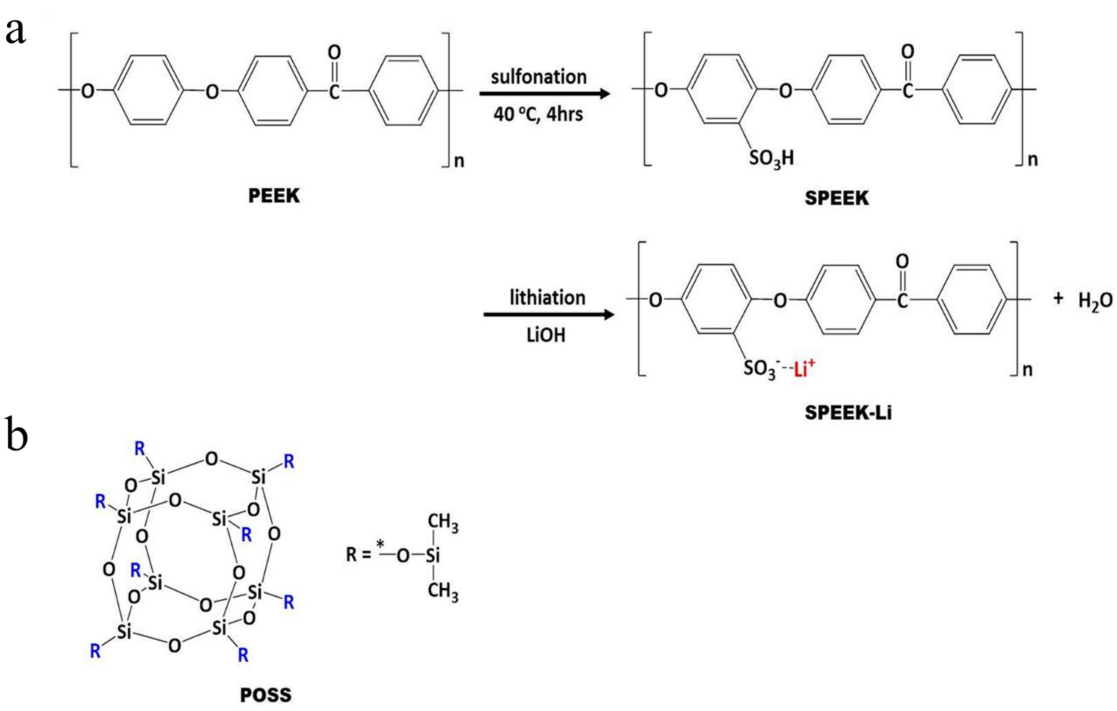

Supplementary Figure 1. (a) Synthesis of SPEEK-Li. (b) Structure of Octakis(dimethylsilyloxy) substituted POSS.

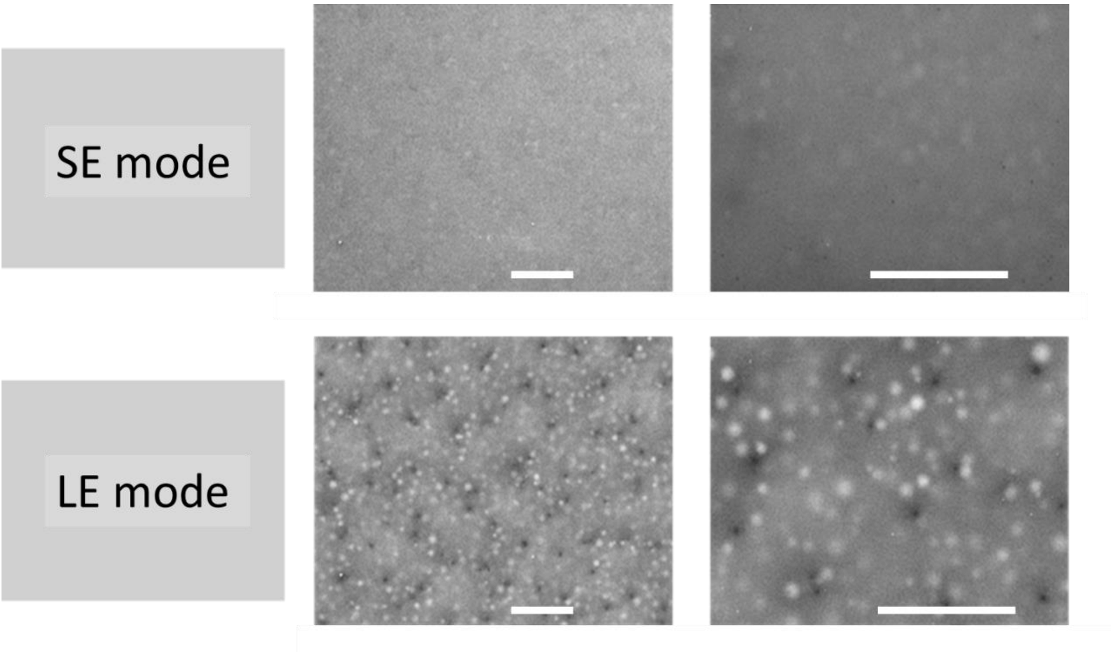

Supplementary Figure 2. Secondary electron (SE) images and low secondary electron (LE) images of SEM micrographs on the top view of the SPEEK-Li/POSS coating. The SE images show the morphology of the very top surface, while the LE images give the structural information deeper below the surface. The images indicate uniformly dispersed POSS clusters embedded within the polymer matrix. (Scale bar: 10 μm.)

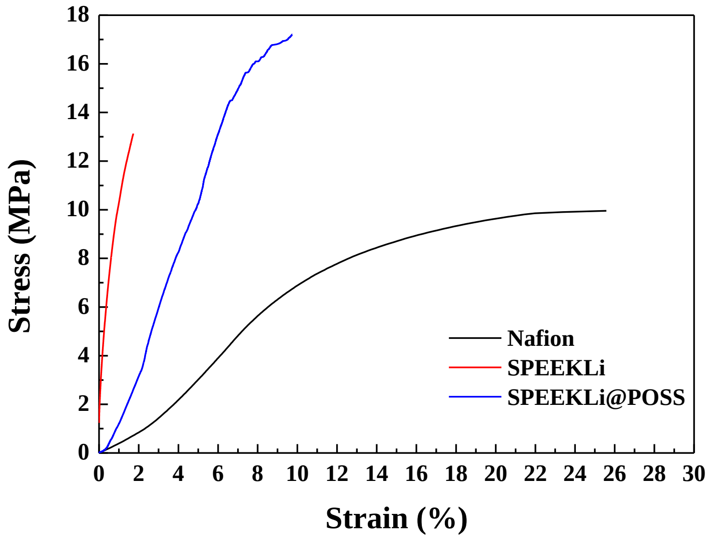

Supplementary Figure 3. Comparison in stress-strain curve: SPEEK-Li shows a tensile (i.e., fracture) stress of 13.3 MPa, which is higher than that (9.9 MPa) of Nafion. SPEEK-Li has an average Young's modulus of 0.9 GPa (for ε= 0.1~0.2%), which is ten times greater than that (0.087 GPa) of Nafion. Compared with SPEEK-Li, SPEEK-Li@POSS has a higher tensile strength (17.4 MPa) by nearly 30% and exhibits substantial improvement in flexibility and stretchability with increasing fracture strain from 1.7% to 9.6%.

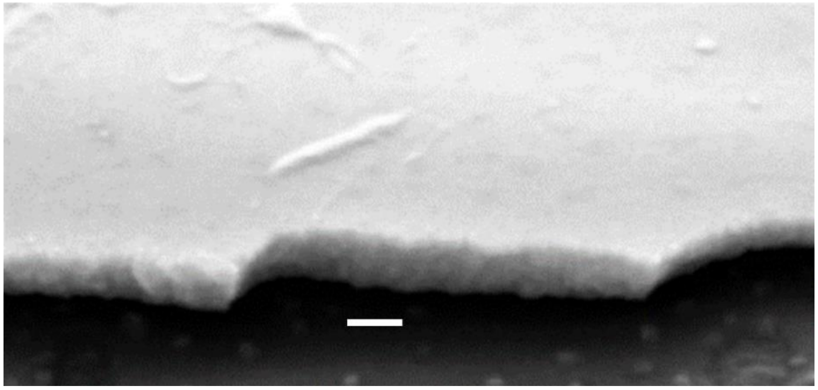

Supplementary Figure 4. SEM image showing cross-section of a SPEEK-Li/POSS membrane coating on Cu electrode. Normally the coating is in close and seamless contact with the Cu substrate. To show the thickness of the coating, the cut electrode sample was twisted for a few times to intentionally create a gap between the polymer coating and Cu substrate. (Scale bar: 100 nm.)

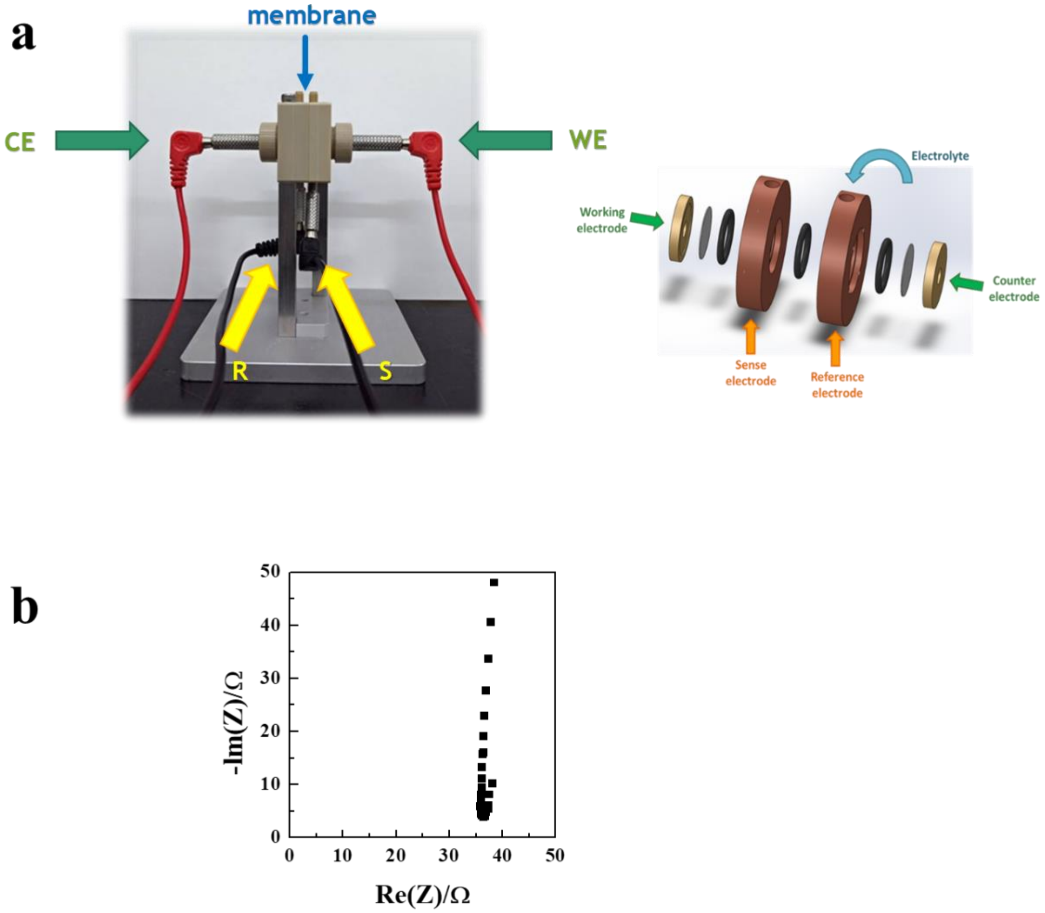

Supplementary Figure 5. The ionic conductivity of the membrane in the electrolyte is determined based on the four-probe measurement design (Supplementary references: 1, 2): (a) A four-electrode double-compartment cell for measuring the ionic conductivity of the membrane using AC impedance. The membrane, along with the four probes are immersed in the electrolyte within the compartment; (b) measured Nyquist plot for SPEEK-Li/POSS membrane

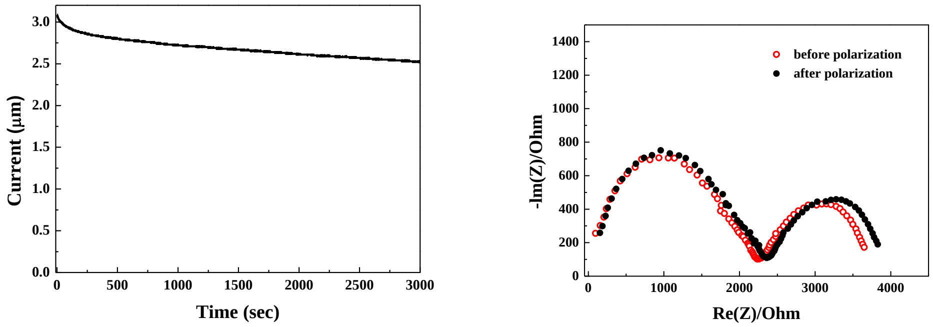

Supplementary Figure 6: Lithium ion transference number (t_{Li+}) was measured based on the Bruce−Vincent method:

$t_{Li+}= I_S (\Delta V-I_0R_i)/ I_0 (\Delta V-I_sR_f])$

where I_0 and I_s are the initial and steady currents in the polarization with a direct-current (DC) voltage of ΔV= 10 mV, respectively. R_i and R_f are the resistance values before and after the perturbation of the system, respectively, as determined by impedance spectra. The SPEEK-Li @POSS membrane was found to possess a t_{Li+} of 0.73.

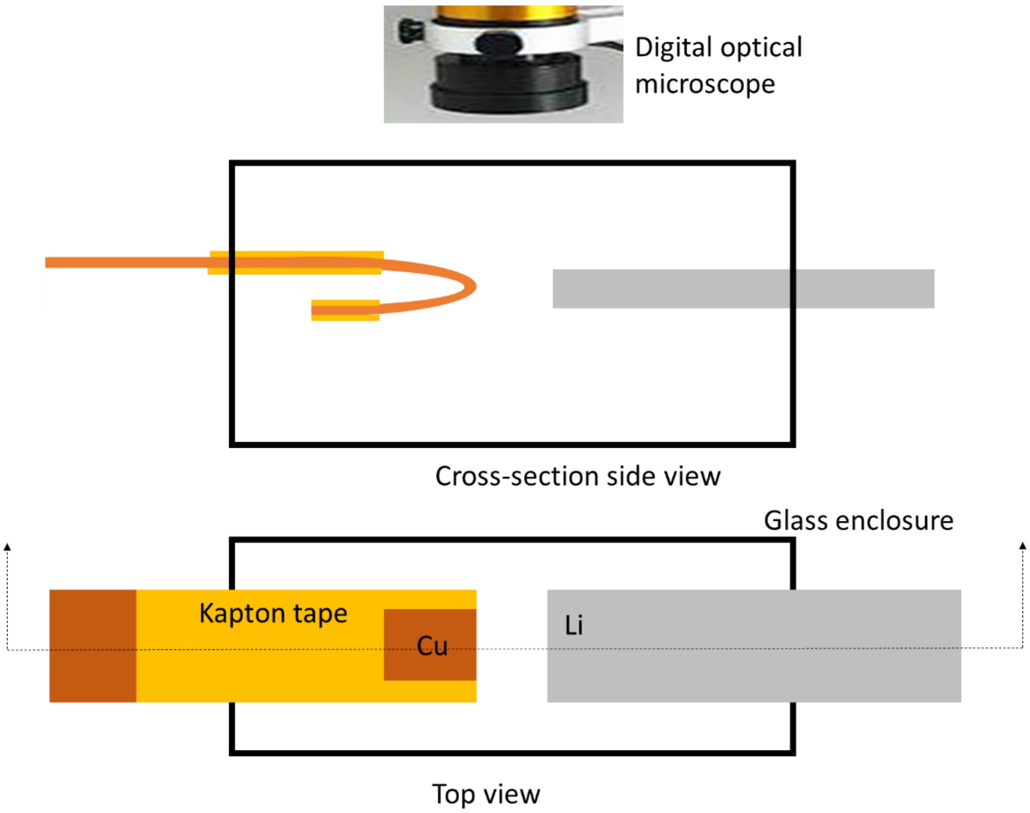

Supplementary Figure 7. Schematics of the glass-cell used for monitoring Li plating-stripping on Cu, with or without SPEEK-Li/POSS membrane coating, in real time.

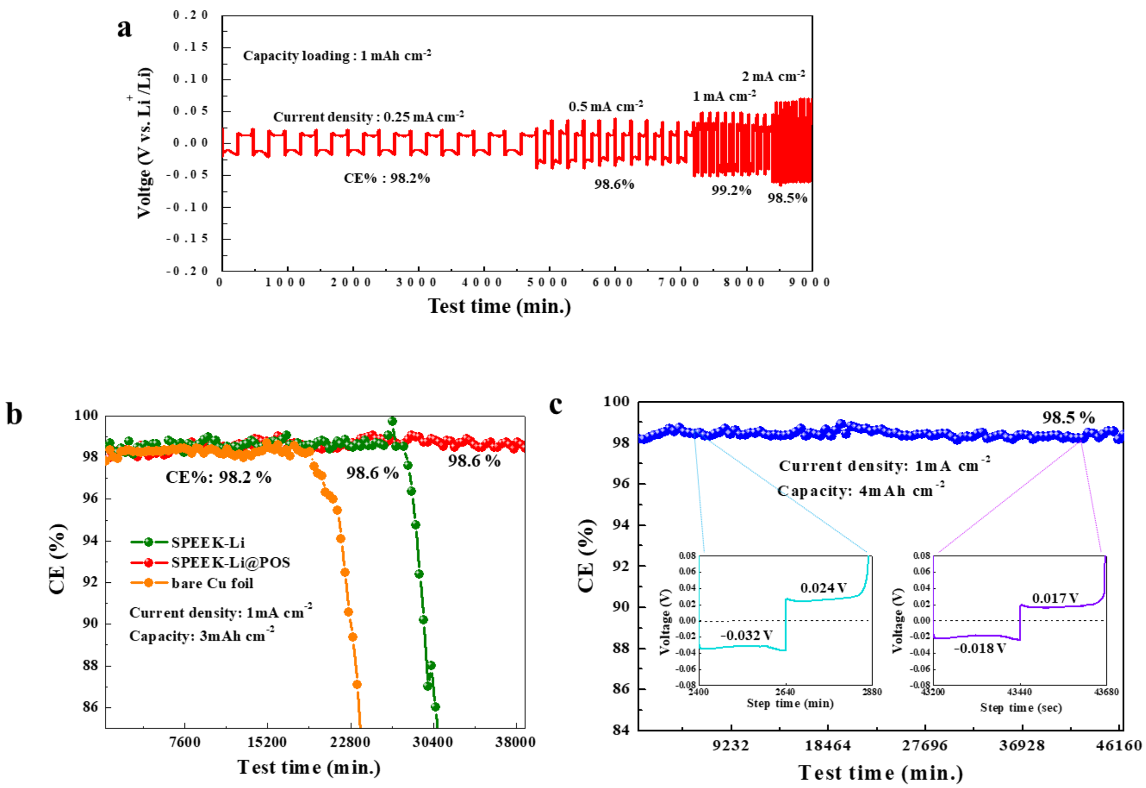

Supplementary Figure 8. Characterizations of electrochemical performance in the ether-based electrolyte 1 M LiTFSI in 1:1=DOL:DME with 3 wt% $LiNO_3$.(a) Voltage profiles of the SPEEK-Li/POSS-coated Cu electrode at current densities of 0.25, 0.5, 1, and 2 mA cm^{-2} for Li-ion loading capacity of 1 mAh cm^{-2}. b) Cycling stability tests of a pristine Cu electrode and SPEEK-Li/POSS-coated Cu electrode at 1 mA cm^{-2} for 3 mAh cm^{-2}. c) Cycling performance of SPEEK-Li/POSS-coated Cu at 1 mA cm^{-2} for 4 mAh cm^{-2}.

Supplementary Table 1: Comparison in coulombic efficiency for different electrolyte/electrolyte modification methods

Current Collector	Method	Electrolyte	CE[e] (%)	Current (mA cm^{-2})	Capacity (mAh cm^{-2})	Cycling hour	Ref.
Cu	SiO_2@PMMA[a] coating	$LiPF_6$ in EC/DEC	90	1	2	200	[3]
Cu	Nano-diamonds additive	$LiPF_6$ in EC/DEC	96	0.5	0.25	20	[4]
Cu	FEC additives	$LiPF_6$ in EC/DEC + 5% FEC	95~ 98	0.1~0.5	0.5	200~ 1000	[5]
Cu	Skin-grafting[b]	$LiPF_6$ in EC: EMC + FEC	98.3	1	0.5	200	[6]
Cu	PDMS[c] coating	$LiPF_6$ in EC/DEC +2% VC	90~ 94.5	0.5~2	1	60~400	[7]
Cu	Cu_3N/SBR[d] coating	$LiPF_6$ in EC:DEC + FEC	97.4	1	1	200	[8]
Cu	Dual salts	LiTFSI + LiBOB in EC:EMC	98.1	0.5	0.5	800	[9]
Cu	atomic layer deposition-Al_2O_3	$LiPF_6$ in EC/EMC	95-98	1	1	360	[10]
Stainless steel	Nano-channel	$LiPF_6$ in EC/DEC	88.6~ 97.6	1~3	0.5	47~240	[11]
Cu	Sn plating	$LiPF_6$ in FEC/EMC	95	0.5	0.5	20	[12]
Cu	SPEEK-Li@POSS coating	$LiPF_6$ in EC:DMC + FEC	97.6~ 98.5	0.25~1	1~3	630~690	this work

[a]PMMA: Poly(methyl methacrylate)

[b]Surface coating with Poly((N-2,2-dimethyl-1,3-dioxolane-4-methyl)-5-norbornene-exo-2,3-dicarboximide

[c]PDMS: Polydimethylsiloxane

[d]SBR: Styrene butadiene rubber

[e]Coulombic efficiency

Supplementary Methods

Simulation. A theoretical model describing the electrodeposition of lithium ions, which can be divided into the bulk electrolyte and membrane regions, was carried out by COMSOL Multiphysics (version 5.2a). In the bulk solution, the mass transport of lithium ions driven by diffusion and migration can be described by[13-15]

$$\mathbf{N}_j = -D_{0,j}(\nabla c_j + \frac{z_j F c_j}{RT}\nabla\phi) \quad (1)$$

Here, N_j, c_j, $D_{0,j}$, and z_j are the ionic flux, molar concentration, the diffusivity in the bulk solution, and the valence of ionic species j, respectively. F, R, T, and ϕ are Faraday constant, the universal gas constant, the absolute temperature, and the electric potential, respectively. In the membrane region, Li^+ ions penetrate and deposit on the electrode surface. The diffusion coefficient in the membrane, however, has a smaller value than that in the bulk electrolyte solution due to the presence of the coated-polymer. Then the mass transport equation goes to

$$\mathbf{N}_j = -D_{eff,j}(\nabla c_j + \frac{z_j F c_j}{RT}\nabla\phi) \quad (2)$$

where $D_{eff,j}(= f \times D_{0,j})$ is the effective diffusion coefficient in the membrane, and is determined by environmental conditions such as solvent, salt concentration, porous entities, and electrostatic interactions between the charged species. We assumed f=1/50 in our modeling that accounts for the drag and steric effects due to the membrane. Note that at the interface of liquid and membrane, the ionic flux $\mathbf{N}_j$, the concentration c_j, and electric potential ϕ are continuous. The conservation of ions in the whole simulated system is expressed as

$$\frac{\partial c_j}{\partial t} = -\nabla \cdot \mathbf{N}_j \quad (3)$$

At the electrode-electrolyte interface, reaction kinetic (i.e., deposition process) on the electrode surface occurs and current density of charge transfer i_{loc} can be typically described by Butler-Volmer equation[13,14]

$$i_{loc} = i_0[\exp(\frac{\alpha F\eta}{RT}) - \exp(-\frac{(1-\alpha)F\eta}{RT})] \tag{4}$$

where i_0, α, and η are the exchange current density, anodic charge transfer coefficient, and overpotential, respectively. We modeled the lithium electrode as an infinite plane with a constant exchange current density i_0=10 mA cm^{-2}. For simplicity, the deformation of the cathode due to the deposition of lithium ions is neglected in our simulation, which is valid when the thickness of the deposition is much smaller than that of electrode. Finer mesh is used on the electrodes to accurately capture the reaction kinetics. Strict mesh-refinement have been tested to ensure all solutions are convergent.

For illustration, an ellipse-like cone is considered with aspect ratio of 6. The distance between two electrodes is 0.1 mm. Note that the computation domain is sufficiently large to avoid affecting the electrochemical phenomenon between the electrodes. An electric potential bias (0.2 V) is applied on the anode while the cathode is grounded (i.e., 0 V). Both of them follow the aforementioned Butler-Volmer equation. Other boundaries are insulated ($\mathbf{n}\cdot\nabla\phi = 0$ and $\mathbf{n}\cdot\mathbf{N}_j = 0$). The initial condition sets the concentration of $LiPF_6$ as 1 M (i.e., $c_{\mathrm{Li}^+} = c_{\mathrm{PF}_6^-} = 1\ \mathrm{M}$). The diffusivity of Li^+ in the bulk solution is assumed as 2×10^{-10} m^2 s^{-1} so the $D_{eff,j}$ is taken as 4×10^{-12} m^2 s^{-1}.[16-18] Other parameters include F=96500 C mol^{-1}, R=8.314 J (mol·K)$^{-1}$, and T=298 K.

Supplementary References

1. Yamada, Y., Iriyama, Y., Abe, T. & Ogumi, Z. Kinetics of lithium ion transfer at the interface between graphite and liquid electrolytes: effects of solvent and surface film. *Langmuir* **25**, 12766-12770 (2009).
2. Busche, M. R. *et al.* Dynamic formation of a solid-liquid electrolyte interphase and its consequences for hybrid-battery concepts. *Nature Chem.* **8**, 426 (2016).
3. Liu, W. *et al.* Core–shell nanoparticle coating as an interfacial layer for dendrite-free lithium metal anodes. *ACS Cent. Sci.* **3**, 135-140 (2017).
4. Cheng, X. B. *et al.* Nanodiamonds suppress the growth of lithium dendrites. *Nat. Commun.* **8**, 336 (2017).
5. Zhang, X. Q., Cheng, X. B., Chen, X., Yan, C. & Zhang, Q. Fluoroethylene carbonate additives to render uniform Li deposits in lithium metal batteries. *Adv. Funct. Mater.* **27**, 1605989 (2017).
6. Gao, Y., Zhao, Y., Li, Y. C. Huang, Q., Mallouk, T. E. & Wang, D. Interfacial chemistry regulation via a skin-grafting strategy enables high-performance lithium-metal batteries. *J. Am. Chem. Soc.* **139**, 15288 (2017).
7. Zhu, B. *et al.* Poly (dimethylsiloxane) thin film as a stable interfacial layer for high-performance lithium-metal battery anodes. *Adv. Mater.* **29**, 1603755 (2017).
8. Liu, Y. *et al.* An artificial solid electrolyte interphase with high Li-ion conductivity, mechanical strength, and flexibility for stable lithium metal anodes. *Adv. Mater.* **29**, 1605531 (2017).
9. Li, X. *et al.* Dendrite-Free and Performance-Enhanced Lithium Metal Batteries through Optimizing Solvent Compositions and Adding Combinational Additives. *Adv. Energy Mater.*

8, 1703022 (2018).

10. Chen, L. *et al.* Lithium metal protected by atomic layer deposition metal oxide for high performance anodes. *J. Mater. Chem. A* **5**, 12297 (2017).
11. Liu, W., Lin, D., Pei, A. & Cui, Y. Stabilizing lithium metal anodes by uniform Li-ion flux distribution in nanochannel confinement. *J. Am. Chem. Soc.* **138**, 15443-15450 (2016).
12. Zhang, S. S., Fan, X. & Wang, C. A tin-plated copper substrate for efficient cycling of lithium metal in an anode-free rechargeable lithium battery. *Electrochim. Acta* **258**, 1201 (2017).
13. Newman, J., Thomas, K. E. Hafezi, H. & Wheeler, D. R. Modeling of lithium-ion batteries. *J. Power Sources* **119**, 838-843 (2003).
14. Danner, T., Singh, M., Hein, S., Kaiser, J., Hahn, H. & Latz, A. Thick electrodes for Li-ion batteries: A model based analysis. *J. Power Source* **334**, 191-201 (2016).
15. Martinez-Rosas, E., Vasquez-Medrano R. & Flores-Tlacuahuac, A. Modeling and simulation of lithium-ion batteries. *Computers & Chemical Engineering*, **35**, 1937-1948 (2011).
16. Nyman, A., Behm, M. & Lindbergh, G. Electrochemical characterisation and modelling of the mass transport phenomena in $LiPF_6$–EC–EMC electrolyte. *Electrochim. Acta* **53**, 6356-6365 (2008).
17. K. Hayamizu, Temperature dependence of self-diffusion coefficients of ions and solvents in ethylene carbonate, propylene carbonate, and diethyl carbonate single solutions and ethylene carbonate + diethyl carbonate binary solutions of $LiPF_6$ studied by NMR. *J. Chem. Eng. Data* **57**, 2012-2017 (2012).
18. Ehrl, A., Landesfeind, J., Wall, W. A. & Gasteiger, H. A. Determination of transport

parameters in liquid binary lithium ion battery electrolytes I. Diffusion coefficient. *J. Electrochem. Soc.* **164**, A826-A836 (2017).
